# How and Why Parents Guide the Media Use of Young Children

**DOI:** 10.1007/s10826-015-0144-4

**Published:** 2015-02-24

**Authors:** Peter Nikken, Marjon Schols

**Affiliations:** 1 Erasmus University Rotterdam, M8-43, PO Box 1738, 3000 DR Rotterdam, The Netherlands; 2 Erasmus School of History, Culture and Communication, Erasmus University Rotterdam, Rotterdam, The Netherlands

**Keywords:** Young children, Television, Educational gaming, Touchscreens, Parental mediation, Media skills, Child development

## Abstract

Children use electronic screens at ever younger ages, but there is still little empirical research on how and why parents mediate this media use. In line with Vygotsky’s zone of proximal development, we explored whether children’s media skills and media activities, next to parents’ attitudes about media for children, and several child and parent-family characteristics, predicted parental mediation practices. Furthermore, we investigated children’s use and ownership of electronic screens in the bedroom in relationship to the child’s media skills. Data from an online survey among 896 Dutch parents with young children (0–7 years) showed that children’s use and ownership of TV, game consoles, computers and touchscreens, primarily depended on their media skills and age, not on parent’s attitudes about media for children. Only touchscreens were used more often by children, when parents perceived media as helpful in providing moments of rest for the child. In line with former studies, parents consistently applied co-use, supervision, active mediation, restrictive mediation, and monitoring, depending on positive and negative attitudes about media. The child’s media skills and media activities, however, had stronger relationships with parental mediation styles, whereas age was not related. Canonical discriminant analysis, finally, captured how the five mediation strategies varied among infants, toddlers, pre-schoolers, and early childhood children, predominantly as a result of children’s media skills, and media activities, i.e., playing educational games and passive entertainment use.

## Introduction

Several literature reviews (e.g., Singer and Singer 2011) have described the young child’s gradual development as a media consumer, i.e., how the child processes media content and handles the devices. Despite their lack of essential perceptual and symbolic understanding and fine motoric skills, even infants are already attentive to media content that matches their social ideas, expectations, and capacities to interpret those media (Barr et al. 2008; Valkenburg and Vroone 2004). Although younger children often experience difficulties in using apps on smart mobile devices, which includes uncontrolled swiping, tapping icons incorrectly, accidentally exiting the app and/or not being able to hear audible gaming instructions, many of them still are motivated to continue to use the device (Chiong and Shuler 2010). Moreover, through age 7, children are also honing their fine-motor skills, which makes it gradually easier for them to manipulate touchscreens, small keys, gadgets and controllers. In addition, young children also become increasingly adept at using symbols, playing pretend games, interpreting relevant cues in their social environment, and gain knowledge of story grammar, which is essential for the formation of interpretive schema for processing more demanding media content. By means of their improved skills, 5–8 year old children have developed a distinct preference for educational games or games that foster competition, because these content types match their developmental status (Priewasser et al. 2012). Also, these older children fluidly move between virtual and real worlds when they are consciously involved in creative practices (Marsh 2010).

Although many children turn into relatively competent users of technologies already at a young age, parents or other caregivers are still facilitators, teachers, and gatekeepers of young children’s media use (Chiong and Shuler 2010). The importance of parents for children’s media practices, which determines their media induced learning, play, and social development, has been addressed in numerous studies on parental guidance. These studies point to several types of guidance, largely described as ‘parental mediation’, which Warren (2001, p. 212) defined as ‘any strategy parents use to control, supervise or interpret media content for children’. In accordance with Vygotsky’s (1986) theory on child development, parental mediation is seen as a key strategy in developing children’s skills to use and interpret the media, foster positive outcomes and prevent negative effects of the media on children. Physical, emotional and social experiences, such as media use, and social interactions related to these activities with parents and siblings, provide a scaffold for the child’s development, especially when they occur within the child’s zone of proximal development (Vygotsky 1978). With regard to young children’s media use, this means that when the child is engaged in specific media activities, the parent should apply a form of mediation that is developmentally appropriate (Schofield Clark 2011).

Parents vary widely in their mediation practices, both in the types of strategies and in the frequency that these strategies are applied. As Ito et al. (2010) noticed, some parents deliberately craft a specific media space at home, for example, by limiting the amount of electronic screens in the house in favor of free play and creative activities. In that vein, some parents facilitate specific media platforms over others because of its educational value, use specific electronic screens as a reward for good behavior (Chiong and Shuler 2010), or select specific digital devices that offer them opportunities to engage deeply in shared play and learning with their children (Takeuchi 2011). In contrast to deliberately creating an ‘educational’ environment, parents may also value media devices for the child’s relaxation and entertainment. As such, parents, for example, pass their mobile devices back to their young children at home or when waiting at shopping malls or on the subway (Chiong and Shuler 2010). Some parents take this even a step further and provide their young children with their own media sets in their bedrooms for reasons of relaxation or other practical gains: parents can then watch their own shows, keep the child occupied so that the parent has time for him/herself or to help the child fall asleep (Haines et al. 2013; Takeuchi 2011; Vaala and Hornik 2014).

In addition to crafting specific domestic media spaces for children, parents also apply various routines in guiding children’s media use (Ito et al. 2010). Several studies have shown that these routines can be divided into distinct types of parental mediation (e.g., Böcking and Böcking 2009; Nikken and Jansz 2006, 2013; Sonck et al. 2013; Valkenburg et al. 1999). The following styles of mediation have been discerned for television and games: (1) posing restrictions on time and content, usually referred to as restrictive mediation; (2) discussing content and giving explanations or instructions to the child to enhance safety, raise critical awareness, or stimulate learning outcomes (active mediation); and (3) co-using the media intentionally with the child together, mostly for entertainment or educational purposes. In today’s mobile media environment, parents also choose (4) supervision as a form of mediation, i.e., staying nearby to keep an eye on the child when it is using an electronic screen on its own, or (5) monitor the child’s online activities afterwards, e.g., checking the browser history or logs from social media applications. Finally, with contemporary electronic devices, parents can also (6) use technical restrictions, such as ‘parental controls’ provided by media devices to regulate or block inappropriate content, although parents prefer the first five social strategies as compared to the use of these technical applications (Livingstone and Helsper 2008).

Previous studies have demonstrated that the extent to which parents guide their children’s media use and which strategies they apply are related to demographic variables, such as the parent’s age, gender, and education or income level. In addition, the parent’s own media use and skills, and family context variables, such as family size, marital status, and the number of media screens at home are important too (e.g., Böcking and Böcking 2009; Valkenburg et al. 1999; Van der Voort et al. 1992). These factors define the circumstances for the parent’s involvement in child raising and the allocation of time and effort to the guidance of their children’s media use (Warren 2003, 2005). Child-rearing labor in many families, for example, is gender-stereotypically divided, with mothers more engaged in most mediation practices (Craig 2006). Also, higher-educated families and higher income families can afford to buy the latest electronic devices as an investment in their child’s intellectual development, and guide the use of these screens more easily than lower-educated and lower income parents (Ito et al. 2010). In large families, parents may find less time to mediate their children’s media use (Van der Voort et al. 1992), though others did not replicate this finding (Nikken and Jansz 2006). Furthermore, parents who are less skilled in using media themselves may find it more difficult to install parental controls on the devices, or to discuss the media content critically with their children as compared to media literate parents (Austin 1993; De Haan 2010). In addition, more media devices at home, in particular when they are placed in the child’s bedroom, may make it more difficult for the parent to supervise these screens and effectively guide the child’s media use (Nikken and Jansz 2013).

Since children nowadays start using media at an early age, particularly parents of young children are expected to consider the value of media for the development of their children and adept their mediation to these values (American Academy of Pediatrics 2001, 2011; Australian Government 2014). Parental mediation research has convincingly shown that parents vary their mediation strategies in accordance with their views on various effects of media content on children. Parents who are concerned about risks and harm more often try to protect their children by monitoring, applying restrictions on media use, supervising the child, and by critically talking to the child about media content, whereas parents who feel that the media offer educational or entertainment opportunities more often co-use the media with their child or actively discuss the content (e.g., Sonck et al. 2013; Valkenburg et al. 1999; Warren 2003). Parental mediation research so far, however, has had little attention for the implicit or explicit considerations that parents may have beyond specific effects induced by media content. These considerations are, for instance, whether media fit at all in the young child’s life (Spitzer 2012), whether children should rather spend time on other non-media related activities (Takeuchi 2011), or whether media should match the child’s capabilities (Chiong and Shuler 2010). It is known, however, that parents may regard children’s media use as a break for themselves or to keep the young child at rest (Haines et al. 2013; Vandewater et al. 2007). Moreover, parents who value this instrumental function of TV sets, i.e., they consider media devices handy to sooth children, are more apt to let their young children watch television or DVD’s for longer periods per day (Vaala 2014).

Finally, parental mediation research also has shown that parents adjust their guidance practices to the child’s age, as well as to the child’s media activities. Parents with children between 0 and 8 years, for example, primarily apply supervision and co-use mediation styles to younger children, whereas with older children these parents increasingly use active and restrictive mediation or monitoring (Nikken and Jansz 2013). Furthermore, during co-viewing of television shows or reading digital books, parent–child interactions vary with the age of the child, as parents integrate objects, characters, and actions that appear on screen with the actual real life experiences of that child (Kim and Anderson 2008; Lemish 1987). Finally, parents apply active mediation mostly in regard to educational television programs, websites and social media applications, and they apply restrictive mediation more often when the child is interested in inappropriate types of content (Cranmer 2006; Küter-Luks et al. 2011; Lee and Chae 2007; Sonck et al. 2013).

Many parents feel that through media use their young children develop, in accordance with their physical, cognitive and emotional capacities, a wide range of media skills, defined as the child’s knowledge and understanding of the role of media and technology in society (Marsh et al. 2005). As far as we know, the relationship between parental mediation and the media skills of young children has not yet been thoroughly empirically investigated. From previous studies on parental views about media effects and their children’s age and media use, it appears that there may be a link with the development of the child’s media skills as well. Especially very young children can be assumed to have limited media-literacy and therefore more susceptible to negative media effects according to their parents. This results in the application of more restrictions in young children’s media use or in more supervised media use and co-use. Chiong and Shuler (2010) indeed noted that adults keep young children motivated to use apps by providing scaffolding and extra prompts for the child to understand media material. In other words, the child’s capacities to use media from the perspective of the parents appears to be an important predictor for the parent’s guidance apart from the child’s actual age. This assumption is grounded in the fact that not all children develop exactly in the same pace in the formative years of their lives.

### The Present Study

This study among a sample of Dutch parents with children aged 0–7 years is focused on how the young child’s media use and their parents’ guidance practices are related to (a) family-parental characteristics, including the parent’s considerations about media in the child’s life, and (b) children’s characteristics, including the child’s media activities, and the child’s age and capacities to use digital media. Since this is an explorative study, we formulated the following three research questions. First, how are parental attitudes about media for children and children’s media skills associated with the use of media devices by young children and with their access to the devices in their bedrooms? (RQ_1_) Second, to what extent can differences in parental mediation styles be explained by parental attitudes about media for children and by the child’s media skills and media activities? (RQ_2_) Finally, to what extent are children’s media skills and activities and parent’s mediation practices related to the child’s development? (RQ_3_) By answering each of these questions we take several parent-family contexts and children’s demographics into account.

## Method

### Participants

In the spring of 2013, an online survey was answered by 1,001 parents living in the Netherlands with one or more children of 0–7 years old at home, who were members of a large online panel. Quotas on the child’s age and parent’s gender were used to arrive at a fairly equal distribution of respondents on these two characteristics. After inspection of the demographics, 105 respondents were excluded from the data set, because they showed inconsistencies regarding the presence of children living at home, the child’s age or because data about the level of income were missing. An investigation of the missing data showed that they were at random, making list wise deletion a suitable option (Allison 2009). The final sample contained 896 parents (see Table 1).

**Table 1:** Descriptive statistics of the dependent and independent variables (*N* = 896)

	Range	Mean	SD
Media devices at home			
TV sets	0–1	.98	.13
Game devices	0–1	.76	.43
Computers	0–1	1.00	.03
Touchscreens	0–1	.91	.29
Media devices in child’s bedroom	0–4	.21	.57
Time spend on media by children			
TV sets	0–195	51.37	41.62
Game devices	0–120	10.53	18.93
Computers	0–120	11.82	18.71
Touchscreens	0–135	12.31	18.02
All media	0–210	83.32	58.87
Time spend on media by parents	0–345	197.21	88.50
Media activities			
Entertainment	1–5	1.91	.72
Educational games	1–5	2.23	.88
Social media	1–5	1.11	.39
Action games	1–5	1.53	.73
Media skills	1–4	2.14	.82
Attitudes about media for children			
Positive effects	1–5	3.55	.65
Negative effects	1–5	3.69	.84
Pacifying	1–5	3.04	.72
Too complicated	1–5	2.47	.79
Parental mediation strategies			
Supervision	1–5	3.09	1.26
Co-use	1–5	2.54	.93
Active mediation	1–5	2.38	.90
Restrictive mediation	1–5	2.53	1.00
Technical restrictions	1–5	2.01	1.05
Demographics			
Gender parent (0 = father)	0–1	.47	.50
Education level parent	1–6	3.92	1.40
Family income	1–5	3.12	1.17
Family size	2–6	3.84	.87
Gender child (0 = boy)	0–1	.49	.50
Age child	0–7	3.42	2.27

Using an online panel has several benefits, specifically cost efficiency, a lower chance of non-response given the immediate interactive question–answer procedure and the guaranteed privacy of the respondents reducing the risk of socially desirable responses (Das et al. 2010). Since almost all Dutch households with children are able to get online, the risk of excluding groups from participation was to some extent reduced (CBS 2013; Schols et al. 2011). Comparing our sample to the Dutch population on their education level and marital status indicated that the respondents are slightly higher educated and more often living together with a partner.

### Procedure

In the online questionnaire, one of the parents was asked to fill in the online questionnaire. The responding parent was asked to answer all questions about their youngest child living at home within the age range of 0–7 years. Answering all questions took on average about 20 min.

### Measures

Table 1 presents an overview of the measures that were used in this study.

#### Access to Media Types

For 10 types of audio-visual media the parents indicated (a) how many of these devices were present at home, (b) whether they were to some extent used by the child, and (c) whether they were accessible in the child’s bedroom. Based on the presence at home and the use by children, we decided to analyze the four media types that were mostly used and present in households: (1) TV sets [TV screens and or DVD/Blue-Ray players]; (2) game devices [controller operated game consoles or handhelds]; (3) computers [mouse or keyboard operated laptops or PC’s]; and (4) touchscreens [tablets like iPads or smartphones]. Two types of devices were excluded from the analyses, because they were hardly present and little used by the children: regular cell phones (used by only 19 % of the children) and e-readers (used by 5 %).

The access to media types in the child’s bedroom varied from 0 = no access to 4 = all four media types present in the child’s bedroom.

#### Use of Media

For each of the 10 media devices that were present at home, the parents reported the average time per day that they and their child use that device. Both for parents and for children the use of a device was set to 0 min if the device was not at home. Several parents indicated a relatively high number of minutes that their children spend on different media. To reduce the influence of outliers, scores were recalculated if they exceeded three times the standard deviation (SD) to a round number of minutes equal to the first quarter of an hour that exceeded three times the SD [e.g., an outlier exceeding a limit of 3 × 29 (= 87 min) was recoded as 90 min; cf. Kline (2011)]. Next, the time that children spend on each of the four types of media devices we defined above was calculated by summing the amount of minutes per device that defined that type of medium. For parents a total score was calculated by summing the amount spend on all four types of devices.

#### Children’s Digital Media Activities

Following Nikken and Jansz (2013), the parents indicated on a five-point scale (ranging from 1 = never to 5 = very often) whether the child used the above indicated computers, game devices or tablets for 18 types of activities. A factor analysis using the Oblimin function (δ = 0.0) on these activities resulted in an ambiguous 2-factor solution. However, an acceptable solution of four factors appeared, after deleting in consecutive factor analyses 6 activities. The deleted items presented activities that are rather difficult for young children (e.g., searching information on the internet, manipulating photos or movies, making phone calls, having video contact/skyping) and, consequently, hardly engaged children. By averaging the data of the applicable items, we constructed the following scales: (1) educational gaming [4 items: memory games; educational math or word games; puzzle games; drawing games; Cronbach’s *α* = .82]; (2) passive entertainment [3 items: watching YouTube; listening to digital stories; using music applications; Cronbach’s *α* = .60]; (3) action gaming [2 items: shooting games; adventure games; Pearson’s *r* = .53, *p* < 0.001]; and (4) social media activities [3 items: chatting; using social media; using SMS, WhatsApp or Ping; Cronbach’s *α* = .86].

#### Children’s Skills in Media Use

For 11 types of handling electronic media devices, the parents indicated on a four-point scale ranging from 1 = not applicable at all to 4 = fully applicable to what extent that type of behavior described their child. The 11 performances varied in difficulty, e.g., ‘observes passively what others do on a device’, ‘is capable in realizing sounds or actions on the screen’, ‘can find certain websites on the internet by him/herself’, ‘knows how to start a game or application by itself’, and ‘is capable of closing pop-ups or other unwanted screens by him/herself’. Principal component analysis using the Oblimin function (δ = 0.0) indicated that parents perceived two types of capacities. Eight items described the child as a self-reliant user of electronic media (Cronbach’s *α* = .93). The other three items described the child as a dependent user of media: ‘needs help from others when using digital media’; ‘explores media at random’; ‘observes passively what others do on a device’. Since these three items did not represent a media-literacy skill, and since the reliability of the construct was rather low (Cronbach’s *α* = .57) these items were not further used in the analyses.

#### Parental Attitudes About Media for Children

Using a five-point scale (ranging from 1 = fully disagree to 5 = fully agree), parents gave their opinion on 22 statements about media and young children. The statements related to both positive and negative effects that media content might have on children and to the role of media in the life of children in a broader sense. The effect statements were based on former studies on parental mediation (e.g., Nikken and Jansz 2006, 2013; Valkenburg et al. 1999). The other statements were derived from studies by Vandewater et al. (2007) and Takeuchi (2011) and from opinions generally encountered in public debates about children and digital media.

Principal component analysis using the Oblimin function (δ = 0.0) resulted in four factors. Four items were removed, as they showed double loadings and theoretically did not fit the four constructs. By averaging the scores of the items that defined the factors, the following scales were constructed: (1) positive media effects [8 items; e.g., screen media help my child to learn; media can teach my child English, electronic media will be good for my child’s school performances; Cronbach’s *α* = .89]; (2) media function as a pacifier [4 items: digital media give a moment of rest for my child; media are a good pacifier for my child; media make my child calm and peaceful; with media my child doesn’t have to be bored; Cronbach’s *α* = .75]; (3) negative media effects [4 items: digital media let my child see or do inappropriate things; media brings my child in contact with wrong people; I’d rather see my child play with other things than digital media; digital media are not as good as normal toys for my child; Cronbach’s *α* = .68]; (4) media are too complicated [2 items: media are too complicated for my child; media do not match with my child’s interests; Pearson’s *r* = .48, *p* < 0.001].

#### Parental Mediation

Parental mediation was measured by asking the parents how often they would apply 17 types of guidance on their child’s media use on a 5-point scale (ranging from 1 = never to 5 = always). Although the items referred to the six types of mediation that have been established in former research on parental mediation of digital media (Nikken and Jansz 2013; Sonck et al. 2013), a principal component analysis using the Oblimin function (δ = 0.0) provided a solution with only two types of mediation: (a) telling about safety; monitoring; and using controls or filters, and (b) applying all other types of behavior. Further analyses with a forced 5-factor solution proved to be in line with the theory on parental mediation, although a few items had some marginal double loadings. Based on the solution in this 5-factor analysis we constructed the following scales by averaging the scores of the defining items: (1) co-use [4 items: using the media together either because the child wants to; or because the parent wants to; using a media device together with the child for fun or entertainment; suggesting the child to use an interesting game, website or app which the parent likes; Cronbach’s *α* = .91]; (2) supervision [2 items: being in the child’s neighborhood when it uses a screen; keeping an eye on the child when it uses media; Pearson’s *r* = .70, *p* < 0.001]; (3) active mediation [5 items: complimenting the child when he or she makes good use of a device; telling the child how to use electronic media properly; telling the child how to be safe on the internet; telling the child what is ‘good’ in an electronic media production; having a conversation with the child about nice or interesting electronic media content; Cronbach’s *α* = .86]; (4) restrictive mediation [3 items: telling the child which websites or games are allowed; telling the child to stop when he or she is using a device too long; allowing the child to use a specific app, game or website which the child picked; Cronbach’s *α* = .76]; and (5) technical restrictions [3 items: using a filter to keep the internet safe; controlling the child’s media behavior afterwards; setting parental controls to manage the child’s media use; Cronbach’s *α* = .80].

#### Demographics

Parents reported the number of persons living at home, and the age and gender of their youngest child and their own (parent’s age varied from 18 to 63 years; M = 37.3, SD = 6.1). Family income was measured on a five-point scale: 1 = less than € 33.000; 2 = about € 33.000 (modal); 3 = between € 33.000 and € 66.000; 4 = about € 66.000; and 5 = more than € 66.000 euro. Parental education was measured on a scale from 1 = no education/primary school to 7 = University Masters degree or PhD.

### Data Analyses

Regression analyses in SPSS 20.1 were used to determine relationships between children’s media use and their parents’ guidance practices, on the one hand, and demographics, family-parental characteristics, including the parents’ attitudes about media in the child’s life, and child factors, such as the child’s media activities, and the child’s capacities to use digital media, on the other hand. Since a stepwise or hierarchical regression analysis did not show any additional insights, only the final regression models are presented.

## Results

Practically all children of our respondents have the opportunity to use electronic screens at home (see Table 1). Only game devices are absent in every fourth household, in particular among families with children aged 0–1 and 2–3 years, compared to families with older children, *F*(3,892) = 15.93, *p* < .05.

On average, young children also do not have many electronic screens in their bedrooms. Only 16 % of the parents reported that their child had 1 or more devices in its room, most often a TV set or a game device. The youngest children, aged 0–1 and 2–3 years, hardly have access to electronic screens in their rooms (about 4.5 % has one media device), whereas older children significantly more often have devices to their disposal, *χ* ^2^ = 133.42, df = 12, *p* < .001. Among the 4–5 year olds 15 % has one device and 3 % has two devices in their room; among the 6–7 year olds 28 % own one device, 7 % two devices, and almost 4 % own three or four devices.

According to the reports of their parents, TV sets are most often used by children. They watch on average about 52 min per day, whereas the other three media types each are used for about 11–12 min on average per day. The youngest children (0–1 years) use both TV sets and touchscreens significantly less than all older children, respectively *F*(3,877) = 38.37, *p* < .001 and *F*(3,810) = 11.87, *p* < .05. The use of game devices and computers significantly increases gradually with age. These devices are used the least per day by 0–1 year olds, somewhat longer by 2–3 year olds, then even significantly longer per day by 4–5 year old children, and finally the longest by 6–7 year old children, *F*(3,675) = 50.11 and *F*(3,891) = 35.08 respectively, *p* < .05.

Table 2 shows the relationships found in multiple regression analyses between the time that young children devote to electronic screens and the number of devices in the bedroom, on the one hand, and parental attitudes about media for children, and parent-family, and child predictors, on the other hand (RQ_1_). Collinearity diagnostics indicated that all variables had a unique contribution.

**Table 2:** Prediction of children’s time spend on electronic media, and of the presence of media devices in the child’s bedroom (standardized coefficients)

	Time spend on				Media devices in bedroom
	TV sets^a^	Game devices^a^	Computers^a^	Touchscreens^a^	
Parent-family variables					
Parental attitudes about media					
Positive effects	−.04	−.03	−.01	−.06	.00
Negative effects	.04	.04	−.03	−.02	−.02
Pacifying	.06	.05	.03	.21***	.08*
Too complicated	−.01	.07	−.01	−.05	.05
Parent’s media use	.31***	.14***	.09**	.08*	−.00
Gender (0 = father)	−.02	−.04	−.06	−.04	−.08*
Educational level	−.17***	−.04	−.07*	−.00	−.13***
Family income	−.03	−.03	−.07*	.13***	−.00
Family size	−.01	.02	.02	−.06	−.03
Child variables					
Media skills	.11**	.21***	.30***	.38***	.18***
Gender (0 = boy)	−.06	−.09*	−.01	−.02	.03
Age	.18***	.30***	.12**	−.01	.22***
*F*	22.75***	17.76***	17.17***	18.88***	13.61***
R^2^	.23	.23	.18	.21	.15
df	12,868	12,666	12,882	12,801	12,883

^a^ Regression analysis applied to the subsample of parents with the media device at home

* *p* < 0.050; ** *p* < 0.010; *** *p* < 0.001

As the upper part of Table 2 shows, the parents’ attitudes about media for children are not strongly associated with the time that children spend on media or with media in the child’s bedroom. Parental views on positive and on negative effects of the media are not associated with the time spend on media or the presence of devices in the child’s room. The same is true for parents’ opinion that media may be too complicated for young children. However, children spend significantly more time with touchscreens and have more electronic screens in their bedrooms when their parents agree that the media provide a moment of rest.

With regard to parent and family variables, the parent’s own media use is an important predictor. Children spend more time with TV sets, game devices, and to a lesser extent with computers and touchscreens, when their parents use electronic media more often. Furthermore, lower-educated parents significantly more often reported that their children have electronic screens in their bedroom and that their children spend more time on watching television and using the computer. Parents with a higher income reported that their children make less use of computers and more use of touchscreens compared to parents with a lower income. Finally, fathers reported somewhat more often than mothers that their children have media devices in their bedroom.

Among the child characteristics, the skills to use media turns out to be an important predictor of the time that children spend on media and of their accessibility to devices in the bedroom. Children who are, according to their parents, better skilled to operate electronic media have more often digital screens in their bedroom and spend more time on all media devices than less-skilled children. These findings are especially clear for touchscreens and computers. Besides the child’s skills, age is also related to the use of and access to media. As already noted above, older children have more media devices in their bedroom and they spend more time watching television, playing videogames, and using the computer. Finally, boys more often than girls spend time on gaming.

The extent to which parental mediation activities can be explained by the parent’s attitudes and the child’s skills and media activities is presented in Table 3 (RQ_2_). Collinearity diagnostics again indicated that there were no confounding relationships between the predictor variables. The results show that parental attitudes about the effects of media on children are important predictors of the parents’ mediation strategies. Parents who agree with the positive influence of media especially more often apply supervision, co-use and active mediation, whereas parents who are concerned about negative effects more often supervise, restrict and use technical restrictions on the young child’s media behavior. These attitudes about positive and negative effects of media content, however, are not the only opinions related to the parent’s mediation practices. First, the view that media functions as a pacifier for the child is paralleled by more restrictions. Second, parents who are convinced that media are too complicated for their child less often supervise and co-use the media with the child and they more often restrict the child’s media use. Finally, these parents also more often use technical restrictions.

**Table 3:** Hierarchical regressions predicting parental media guidance among parents with young children (standardized coefficients)

	Supervision	Co-use	Active mediation	Restrictive mediation	Technical restrictions
Parent-family variables					
Parental attitudes about media					
Positive effects	.18***	.28***	.25***	.06	.09*
Negative effects	.15***	.03	.03	.12***	.08*
Pacifying	−.04	−.02	−.06	.08*	−.02
Too complicated	−.11***	−.10**	−.04	−.07*	.09*
Parent’s media use	−.03	−.03	−.07*	−.06*	−.02
Gender (0 = father)	.06*	−.00	−.01	.03	−.02
Education level	.03	.04	−.01	.04	−.10**
Family income	−.05	−.03	−.02	.02	−.02
Family size	.08**	.04	.09***	.12***	.13***
Child variables					
Media skills	.11*	.15**	.20***	.27***	.20***
Entertainment	.16**	.15**	.06	.04	.05
Educational games	.18***	.16***	.20***	.15***	.11**
Social media	−.04	−.02	.04	−.00	.12**
Action games	−.05	−.02	.02	.03	−.05
Child’s media use	.03	.07	.04	.07	.01
Media in bedroom	−.07*	−.02	−.01	−.03	.01
Gender (0 = boy)	−.06	.01	−.00	−.02	−.05
Age	.08*	−.02	.10*	.09*	.07
*F*(18,877)	21.66***	28.93***	38.83***	35.85***	14.18***
R^2^	.29	.36	.43	.41	.21

* *p* < 0.050; ** *p* < 0.010  *** *p* < 0.001

The context of media use at home is also influential. With the exception of co-use, parents in larger families more often use all mediation types. Moreover, mothers more often apply supervision than fathers, and lower-educated parents more often use technical restrictions on the child’s media use than higher-educated parents. Finally, parents who spend more time on the media themselves are somewhat less inclined to apply active and restrictive mediation on their child’s media use.

As shown in the lower part of Table 3, the child’s skills to use digital media and the types of media content the child is engaged in are especially important in explaining the differences in parental mediation of children’s media use. Parents apply all types of mediation more often, in particular active and restrictive mediation and technical restrictions, when their child is more skilled in operating the media. Furthermore, especially regarding entertainment content, parents supervise their child’s media use or co-use electronic screens with their child. Moreover, they apply all mediation strategies more often when their child is engaged in educational gaming and apply technical restrictions more often when their child is involved in social media activities. Parents do not apply more or less mediation when the child spends more time on electronic screens, but children who have more devices in their room are less often supervised by their parents. Finally, it also appeared that parents apply somewhat more active and restrictive mediation on older children, regardless of their capacities to use electronic media. Parents, however, do not vary their mediation practices for their sons or daughters.

So far, we analyzed for each mediation style separately how it was associated with parental attitudes, children’s media skills and media activities. In order to get a more concise picture of differences among age groups, a discriminant analysis was performed (RQ_3_). In this analysis, we used the five mediation styles, four parental attitudes about media for children, the children’s media skills and the four types of children’s media activities as discriminant variables. The objective was to find the linear combinations of these variables that best discriminated between the four age groups in our study. Three discriminant functions resulted from the analysis (see Table 4). The canonical correlation of the first and second function was respectively .72 (Wilk’s *λ* = .44; *χ* ^2^ = 729.24, df = 42, *p* < .001) and .29 (Wilk’s *λ* = .90; *χ* ^2^ = 95.30, df = 26, *p* < .001). The canonical correlation of the third function was low: .14 (Wilk’s *λ* = .98; *χ* ^2^ = 16.50, df = 12, n.s.), which indicates that this function is not discriminating. Therefore, we will not discuss it further.

**Table 4:** Canonical discriminant analysis; structure matrix among parents with children in four age groups

Function	I	II	III
Parental mediation styles			
Supervision	.32	**.40**	.01
Co-use	.31	**.40**	.17
Active mediation	**.49**	.12	.01
Restrictive mediation	**.50**	**.55**	−.15
Technical restrictions	.32	−.03	−.13
Parental attitudes about media			
Positive effects	.19	−.10	.18
Negative effects	.14	.10	**−.50**
Pacifying	−.02	.09	.12
Too complicated	−.15	−.11	−.07
Child’s media skills	**.77**	−.04	**.53**
Child’s media activities			
Entertainment	.26	**.43**	**.42**
Educational games	**.59**	**.55**	.04
Social media	.12	−.10	.17
Action games	**.56**	−.22	−.13
Eigenvalue	1.05	.09	.02
Variance (%)	90.3	8.0	1.6

Bold values indicate the defining variables for each function

The first discriminant function was above all defined by the child’s skills to use the media, followed by playing educational and action games and by the parent’s active and restrictive mediation. This function thus represents the dimension of the developing child as a skilled, active user of educational or action based media content and who is primarily guided by active and restrictive mediation. The second function was also defined by the child as a user of educational media who is guided by restrictive mediation, but also by using media for passive entertainment and guided by means of co-use and supervision. This function thus rather stands for the child that uses the media especially together with or under supervision of the parent for education and entertainment. Interestingly, the attitudes of the parent about media use by children did not have high weights in both discriminant functions, indicating that the perceptions about media use by young children do not vary strongly between parents of the youngest and the somewhat older children.

**Fig. 1:**
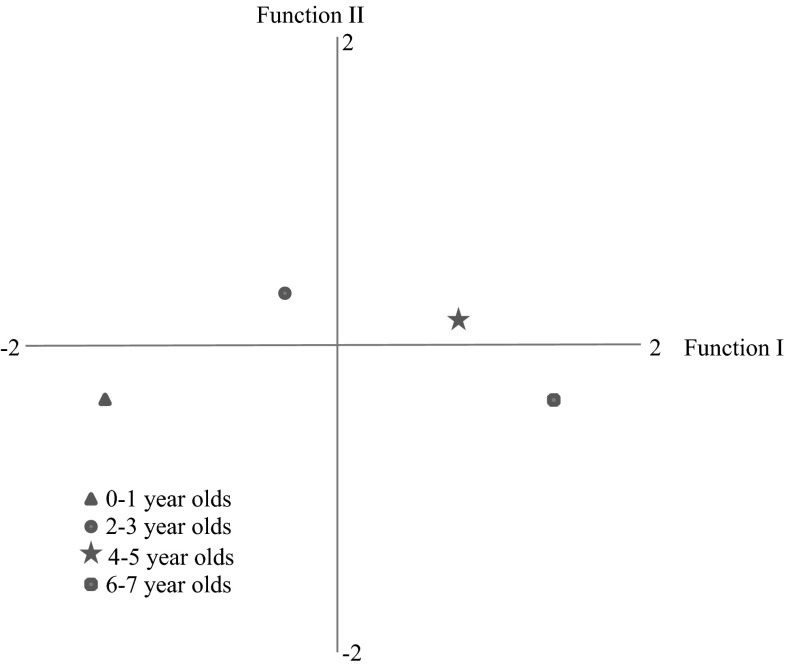
Unstandardized canonical discriminant functions evaluated at group means

The positions of the four age groups, each situated in its own quadrant, are presented in Fig. 1. As can be seen on the horizontal axis, the youngest children (0–1 year old), fitted the least in the profile of the skilled, self-reliant media consumer of educational or action based media and guided by active and restrictive mediation, whereas the oldest children (6–7 year olds) are best described by that profile. The positions of the four age groups on the second dimension (vertical axis) do not vary dramatically, but indicate that both 0–1 year olds and 6–7 year old children have the least in common with the profile of a user of educational or entertaining media together with the parent. Children aged 2–3 years and to a somewhat lesser degree aged 4–5 years are best described by this profile.

## Discussion

Among a sample of Dutch parents with children aged 0–7 years, this study explored to what extent children’s use of media devices at home and children’s access to these screens in the bedroom are guided by parent’s attitudes about media for children and by the child’s skills to use electronic screens. In addition, we analyzed how differences in the mediation practices of the parents can be explained by their attitudes about media for their children, the types of media content these children are engaged in, and the children’s media skills. Finally, we were interested in how the children’s media skills, the types of media content the child is engaged in, and the parent’s mediation practices together are related to the young child’s development. Research on the parent’s influence on young children’s use of technologies is important, because routines in media use by children and cultural tastes and preferences are shaped already at a young age. Moreover, there is still little systematic data on the mediation practices of parents with young children, even though, as the results of our study confirmed, most of these children are growing up in a media-saturated home environment and also use the different types of available media.

Our research questions were guided by Vygotsky’s (1978) theory on the zone of proximal development (ZPD). According to this theory, social agents, i.e. the parents, and tools in the environment, i.e. electronic screens, can both scaffold the child’s development when they match with the child’s ZPD. Therefore, parents are expected to apply developmentally appropriate types and amounts of mediation (Schofield Clark 2011).

The results indicated that when parents support their child in using electronic screens, they indeed adjust the amount and type of their scaffolding activities to the young child’s developing media capacities and media activities. The child’s skills to use electronic screens, as reported by the parents, are an especially important predictor of the parent’s mediation practices as well as of the child’s use of media and access to electronic screens in the bedroom. The skills to use media in a self-reliant manner contributed, in particular, to a higher use of computers and touchscreens and to more supervision, co-use, active and restrictive mediation and more monitoring by their parents. Age, which in most former studies proved to be an important indicator of parental mediation practices (e.g., Valkenburg et al. 1999), did not explain many of the differences in the applied mediation styles. Apparently, there are important differences in the media capacities and the content preferences of the child that do not necessarily parallel the child’s age. Thus, some children may have already achieved particular skills in using the media and developed preferences for a particular type of content, whereas other children of the same age may not yet have achieved these skills or preferences, prompting the parent to mediate differently. Moreover, the child’s skills in handling media devices were also associated with the possession of electronic screens in the bedroom and the amount of time that children spend with these screens. This means that parents not only adapt their behavioral routines and practices to the child’s development as a young media consumer, but they also construct the electronic screen environment in the child’s bedroom according to the child’s capacities to handle these devices.

The discriminant analysis clearly captured the development of children as media users in relation to the parent’s scaffolding of that development. Parents are not very involved in parental mediation among infants, most likely due to the low use of devices by these young children and their lack of skills to use these devices by themselves. Parents mainly apply strategies of co-use and mediation to toddlers’ media use. Though these 2–3 years olds spend time on television and touchscreens and use educational games and entertainment media, they are still rather unskilled media consumers. Children aged 4 years and older are more skilled media consumers, practicing their skills during co-use with the parent or under their direct supervision. These children also progressively get access to their own computers and game devices, spend more time on electronic screens and develop a further interest in educational and action based games and in social media. The oldest children, finally, are seen by their parents as even more self-reliant and capable to use media devices and play educational or even action based games on their own. Parents then decrease their co-use and instead increase their active and restrictive mediation practices. With regard to the young child’s development as a media consumer, we conclude that parents adapt their mediation practices and the provision of media devices in the child’s bedroom, as the child grows from infancy to middle childhood. However, there are large differences in the mediation styles applied per age group depending on the child’s skills to handle the media and the child’s preferences for specific types of content.

From our findings we also infer that parents have a broad view on the role of media for children that goes beyond the risk–benefit paradigm. Besides the positive educational and learning effects of screen devices and the negative effects of media content, parents also take the complexity of the media and the practical value of the media for structuring their family life into consideration when balancing their young children’s media use. Interestingly, the extent to which parents value such considerations did not relate to the time young children spend on the electronic devices or to their access to such screens in their bedrooms. Children do not spend less time behind electronic screens when their parents expect negative outcomes or are more reserved about media use. The only exception here is the view on the practicality of media as a soothing instrument. In line with what Haines et al. (2013), Vandewater et al. (2007) and recently Vaala (2014) already noticed for television sets, we found that young children have more access to several types of electronic screens in their bedroom when their parents regard such devices as a pacifier for the child or instrumental for family routines. Moreover, with regard to touchscreens, our findings also especially corroborate the pass-back effect that was observed by Chiong and Shuler (2010); parents hand over their tablets or smartphones to their young children at various moments during the day intentionally to keep them at rest or busy and to have some time for themselves.

Although the young child’s media use by itself was not associated with the parents’ views on media effects on children, these perceptions are, however, relevant for the frequency and type of mediation that parents use to guide their children’s media use. In line with former parental mediation research on older children (e.g., Valkenburg et al. 1999), concerns about negative effects of media use and positive attitudes towards media effects systematically predicted the parent’s mediation. In addition, it appeared that parents who consider the media too complicated for their child, less often supervise and co-use the media with the child or restrict the child’s media use. These parents do, however, more often use technical restrictions. Perhaps, these parents think that their low-skilled child is not able to access media content when it is shielded from them by means of technical measures. These measures do not require the parents themselves to be actively involved in their child’s media use. Finally, it appeared that parents who value the media as a pacifier to keep the child quiet or as a way to structure family media routines, are not necessarily lenient in their mediation practices. On the contrary, these parents tend to be more restrictive about the time their children use media devices or have access to media-content, which indicates that they still balance the instrumental value of media devices with their vigilance towards children’s safe use of media.

As noted above, parents take into consideration the young child’s engagement in media content and the skills to handle the media and combine this with their own views on the contribution of these media for the child’s development and well-being. Our results also indicated that parents reckon with contextual factors within the home environment. First, in households with more persons at home, parents use all mediation strategies more often with their youngest child, except for co-use. Since we specifically asked about the youngest child at home, we surmise that in these larger families there are older siblings at home. The youngest child may then encounter more different types of media and content and possibly even adapt media preferences and skills in using media. In smaller families, children probably grow up with no or less older siblings and are less likely to encounter media content that is aimed at older children. Therefore, parents with more children may be more often forced to think about the role of the media for the younger child and to apply more mediation than parents with one child. Second, children with parents spending more time on different types of media, also spend more time on media devices and receive less restrictive and active mediation. This implies that parents who are engaged in media use themselves set an example for their children to be more involved in media use too. Third, we found differences among families of different socio-economic backgrounds. Higher-educated parents and those with a higher income appear to structure their young children’s media environment at home by using more often the newest forms of technology compared to lower-educated parents and those with a lower income. Since smart mobile devices are still rather expensive, parents with a lower socio-economic status have fewer opportunities to acquire the latest versions of high-end media products. Consequently, their children also have less opportunities to acquire the skills to use these media. As Paus-Hasebrink et al. (2014) noticed in a review of studies among families with somewhat older children, parents in socio-economically disadvantaged environments often lack skills in using media. Therefore, they are less able to consistently explain their children how media and media systems work and experience difficulties in deliberately scaffolding their children’s media use. Furthermore, rules are set inconsistently, resulting in unclear guidance and contact with the parents. This uncertainty and inconsistency may lead children from low-income households to exploit their parents’ lack of consistency and use any kind of media whenever and wherever they want (Paus-Hasebrink et al. 2014, p. 11). The results of our explorative study indicated that this risk in lower socio-economical families also may be at stake for very young children. Children living in lower-income families make less use of touchscreens in favor of desktop computers or laptops. Furthermore, children from lower-educated parents have more devices to their disposal in the bedroom and spend more time on watching television and using the computer.

The results of our study provide some practical outcomes, both for researchers and practitioners. First, the finding that there are large differences in media use and skills among children of the same age implies that children’s media skills are important to take into consideration when investigating media use among young children and their parents’ involvement with that usage. In this study, we asked primarily about the young child’s technical media skills and competences. In future studies, this interest may be broadened to, for example, motoric skills in handling the mouse or keyboard or tablets, or competences specifically related to understanding media content and formal features, or to interacting with others by means of social media.

Second, from our data it appears that young children spend quite some time on using different types of media. TV is the primary medium; game devices and computers are gradually more used as the child matures. Furthermore, about 1 in 8 children has a device in his or her bedroom, mostly a TV set or a game device. This is not in line with the health advice provided by governments and national institutions (cf. American Academy of Pediatrics 2001, 2011; Australian Government 2014). Since our study indicated that media use is high among young children with media-active parents, professionals who want to support parents in raising young children in a saturated media environment may want to take the parental media use in account when curbing young children’s media use. These findings reflect the parent as a role-model for children, who copy their behavior. Hence, getting young children more into play, out-door activities or sports, may be more successful when parents are aware that their media behavior at home may serve as an unwanted example for their children. In addition, older siblings may also serve as a role model for their younger siblings, making it relevant for professionals to also reckon the presence and age of siblings at home. Finally, family support should be concentrated on what the child is capable of doing with electronic screens, perhaps more than whether the child is younger or older than two years. Especially in the age span of 0–7 years, children rapidly develop, but each child on its own pace. Therefore, guidelines on media use make sense for some parents, whereas other parents may feel that their young child is already up to using screens for entertainment or for learning. Parental support should thus be as tailor made as possible in order to be effective.

### Limitations

There are some limitations to our study that should also be acknowledged. First, the data is reliant on parental reports of the child’s media use and their skills in using the media. Though we did not expect parents to over-report their children’s skills or under-report their media use because of social desirability, we cannot exclude that some parents may have done so. Nevertheless, though parents may find it difficult to report exact time uses and children’s digital skills, this method appears to be the best alternative to investigate how parents guide their young children’s media use and why. These young children cannot report their own use and skills themselves and other methods are very costly. Second, the survey we used was gathered for this study as part of a national campaign on media-literacy. Therefore, the questionnaire did not incorporate an extended set of items for some variables, e.g. children’s media skills. Furthermore, parent’s mediation practices did not optimally mirror the theoretical structure found in previous studies (e.g., Nikken and Jansz 2013). A possible explanation is that young parents with infants or toddlers who formed a substantial part of our dataset have not yet internalized their mediation practices into their belief system. More experienced parents with older children may have more explicit views on their parental mediation behaviors. Although this warrants for further research, the forced factor analysis we used and the regression results fully corroborated the theory on parental mediation. Therefore, despite these limitations, the results provide an interesting indication of how parents of young children guide these children’s media usage and why they do so.

## References

[CR1] Allison PD, Millsap RE, Maydeu-Olivares A (2009). Missing Data. The SAGE handbook of quantitative methods in psychology.

[CR2] American Academy of Pediatrics (AAP), Committee on Public Education (2001). Children, adolescents, and television. Pediatrics.

[CR3] American Academy of Pediatrics (AAP), Committee on Public Education (2011). Media use by children younger than 2 years. Pediatrics.

[CR4] Austin E (1993). Exploring the effects of active parental mediation of television content. Journal of Broadcasting and Electronic Media.

[CR5] Australian Government (2014). Move and play every day: National physical activity recommendations for children 0–5 years.

[CR6] Barr R, Zack E, Muentener P, García A (2008). Infants’ attention and responsiveness to television increases with prior exposure and parental interaction. Infancy.

[CR7] Böcking S, Böcking T (2009). Parental mediation of television: Test of a German-speaking scale and findings on the impact of parental attitudes, sociodemographic and family-factors in German-speaking Switzerland. Journal of Children and Media.

[CR8] CBS (2013). *ICT gebruik van huishoudens naar huishoudkenmerken*. Heerlen/Den Haag: Centraal Bureau voor de Statistiek. http://statline.cbs.nl/StatWeb/publication/?DM=SLNL&PA=71102NED&D1=3&D2=0,2,4-5&D3=l&VW=T. Accessed 1 October 2013.

[CR9] Chiong C, Shuler C (2010). Learning: Is there an app for that? Investigations of young children’s usage and learning with mobile devices and apps.

[CR10] Craig L (2006). Does father care mean fathers share? A comparison of how mothers and fathers in intact families spend time with children. Gender and Society.

[CR11] Cranmer S (2006). Children and young people’s uses of the internet for homework. Learning Media and Technology.

[CR12] Das M, Ester P, Kaczmirek L (2010). Social research and the internet: Advances in applied methods and new research strategies.

[CR13] De Haan J, Ferro E, Kumar Dwivedi Y, Ramon Gil-Garcia J, Williams MD (2010). Late on the curve; causes and consequences of differences in digital skills. Handbook of research on overcoming digital divides: Constructing an equitable and competitive information society.

[CR15] Haines J, O’Brien A, McDonald J, Goldman RE, Evans-Schmidt M, Price S, King S, Sherry B, Taveras EM (2013). Television viewing and televisions in bedrooms: Perceptions of racial/ethnic minority parents of young children. Journal of Child and Family Studies.

[CR16] Ito M, Baumer S, Bittanti M, Boyd D, Cody R, Herr-Stephenson B, Horst H, Lange P, Mahendran D, Martinez K, Pascoe C, Perkel D, Robinson L, Sims C, Tripp L (2010). Hanging out, messing around, and geeking out: Kids living and learning with new media.

[CR17] Kim J, Anderson J (2008). Mother-child shared reading with print and digital texts. Journal of Early Childhood Literacy.

[CR100] Kline, R. B. (2011). *Principles and practice of structural equation modelling* (3rd ed.). New York: Guilford Press.

[CR18] Küter-Luks T, Heuvelman A, Peters O (2011). Making Dutch pupils media conscious: Preadolescents’ self-assessment of possible media risks and the need for media education. Learning Media and Technology.

[CR19] Lee S, Chae Y (2007). Children’s internet use in a family context: Influence on family relationships and parental mediation. CyberPsychology and Behavior.

[CR20] Lemish D, Lindlof T (1987). Viewers in diapers: The early development of television viewing. Natural audiences: Qualitative research of media uses and effects.

[CR21] Livingstone S, Helsper E (2008). Parental mediation and children’s internet use. Journal of Broadcasting and Electronic Media.

[CR22] Marsh, J. (2010). *Childhood, culture and creativity: A literature review*. Newcastle: Creativity, Culture and Education. http://www.creativitycultureeducation.org/childhood-culture-and-creativity-a-literature-review. Accessed May 2014.

[CR23] Marsh, J., Brooks, G., Hughes, J., Ritchie, L., Roberts, S. & Wright, K. (2005). *Digital beginnings: Young children’s use of popular culture, media and new technologies.* Sheffield: University of Sheffield. http://www.digitalbeginnings.shef.ac.uk/DigitalBeginningsReport.pdf. Accessed May 2014.

[CR24] Nikken P, Jansz J (2006). Parental mediation of children’s videogame playing: A comparison of the reports by parents and children. Learning Media and Technology.

[CR25] Nikken P, Jansz J (2013). Developing scales to measure parental mediation of young children’s internet use. Learning Media and Technology.

[CR27] Paus-Hasebrink, I., Sinner, P. & Prochazka, F. (2014). *Children’s online experiences in socially disadvantaged families: European evidence and policy implications*. London: EU Kids Online, LSE. http://www.lse.ac.uk/media@lse/research/ EUKidsOnline/EU Kids III/Reports/Disadvantaged_children.pdf. Accessed 26 June 2014.

[CR29] Priewasser B, Roessler J, Perner J (2012). Competition as rational action: Why young children cannot appreciate competitive games?. Journal of Experimental Child Psychology.

[CR31] Schofield Clark L (2011). Parental mediation theory for the digital age. Communication Theory.

[CR32] Schols M, Duimel M, De Haan J (2011). Hoe cultureel is de digitale generatie? Het internetgebruik voor culturele doeleinden onder schoolgaande tieners.

[CR33] Singer D, Singer J (2011). Handbook of children and the media.

[CR34] Sonck N, Nikken P, De Haan J (2013). Determinants of internet mediation: A comparison of the reports by parents and children. Journal of Children and Media.

[CR35] Spitzer M (2012). Digitale Demenz. Wie wir uns und unsere Kinder um den Verstand bringen.

[CR36] Takeuchi L (2011). Families matter: Designing media for a digital age.

[CR37] Vaala S (2014). The nature and predictive value of mothers’ beliefs regarding infants’ and toddlers’ TV/video viewing: Applying the integrative model of behavioral prediction. Media Psychology.

[CR38] Vaala S, Hornik R (2014). Predicting US infants’ and toddlers’ TV/video viewing rates: Mothers’ cognitions and structural life circumstances. Journal of Children and Media.

[CR39] Valkenburg P, Krcmar M, Peeters A, Marseille N (1999). Developing a scale to assess three styles of television mediation: “Instructive mediation”, “restrictive mediation”, and “social coviewing”. Journal of Broadcasting and Electronic Media.

[CR40] Valkenburg P, Vroone M (2004). Developmental changes in infants’ and toddlers’ attention to television entertainment. Communication Research.

[CR41] Van der Voort T, Nikken P, Van Lil J (1992). Determinants of parental guidance of children’s television viewing: A Dutch replication study. Journal of Broadcasting and Electronic Media.

[CR42] Vandewater EA, Rideout VJ, Wartella EA, Huang X, Lee JH, Shim M (2007). Digital childhood: Electronic media and technology use among infants, toddlers, and preschoolers. Pediatrics.

[CR43] Vygotsky LS (1978). Mind in society.

[CR44] Vygotsky LS (1986). Thought and language.

[CR45] Warren R (2001). In words and deeds: Parental involvement and mediation of children’s television viewing. The Journal of Family Communication.

[CR46] Warren R (2003). Parental mediation of preschool children’s television viewing. Journal of Broadcasting and Electronic Media.

[CR47] Warren R (2005). Parental mediation of children’s television viewing in low-income families. Journal of Communication.

